# Assessment of lipoedema awareness among polish women- online survey study

**DOI:** 10.1186/s12905-023-02614-7

**Published:** 2023-08-29

**Authors:** Rita Hansdorfer-Korzon, Monika Czerwińska, Jacek Teodorczyk, Jolanta Szamotulska

**Affiliations:** 1 Department of Physiotherapy, Medical University of Gdańsk, 7 Dębinki Street, Gdańsk, 80-211 Poland; 2 Department of Nuclear Medicine and Radiology Informatics, Medical University of Gdańsk, 17 Mariana Smoluchowskiego Street, 80-214, Gdańsk, Poland

**Keywords:** Lipoedema, Lipedema, Awareness, Diagnostic challenges, Survey study

## Abstract

**Background:**

Lipoedema is an adipose tissue disorder that is still not fully understood. The primary purpose of this study is to explore the state of knowledge and understanding of lipoedema among Polish women. The secondary aim is to investigate the possible association between knowledge and factors such as BMI, self-reported symptoms, and age.

**Methods:**

One hundred seventy polish women took part in an online survey study that was posted to social media groups and forums in January 2022. The survey consisted of 12 questions and aimed at assessing the basic knowledge about lipoedema.

**Results:**

The least proportion of participants (4%) could correctly indicate the methods of lipoedema treatment. The accurate definition of lipoedema was indicated only by 7% of women, 12% identified characteristic features of lipoedema, and 37% correctly evaluated lipoedema curability. The greatest proportion of respondents knew the differences between obesity and lipoedema (50%).

**Conclusions:**

Awareness of lipoedema among women is deficient. A significant proportion of the respondents report the occurrence of lipoedema symptoms. In order to improve the situation of lipoedema patients, it is crucial to increase the knowledge about this condition both among medical professionals and the general public.

**Supplementary Information:**

The online version contains supplementary material available at 10.1186/s12905-023-02614-7.

## Background

Lipoedema is a complex and problematic disease, both from the medical and the social point of view [1]. It is a relatively unknown condition, characterized by excessive bilateral, symmetrical accumulation of adipose tissue mostly around the lower extremities and, in many cases, also around the upper extremities [2, 3]. The study by Cornely stated that the presence of lipoedema symptoms both in the lower and upper limbs occurs in even up to 90% of lipoedema cases [3]. Patients affected by lipoedema present a noticeable disproportion between the slim trunk and enlarged lower and/or upper limbs [4]. A very characteristic feature of lipoedema is that the feet are not affected, which causes the formation of fat cuffs around the ankles [5]. The disease affects women, and its onset is associated with puberty, pregnancy, or menopause [6]. Some rare cases of lipoedema could be observed in men [7, 8]. Patients affected by lipoedema usually experience such symptoms as heaviness in the lower limbs, pain on pressure, easy bruising, and decreased ability to perform daily activities. These symptoms have a direct impact on their quality of life [9–11]. The course of the disease and the severity of the symptoms varies significantly between individuals [11].

The pathogenesis and etiology are still not fully understood. Following current data, lipoedema is thought to be caused by a combination of genetic factors and hormonal changes [12, 13]. Due to the high percentage of misdiagnosed cases, it is very difficult to accurately determine the exact occurrence of lipoedema, and currently, available data suggest only the estimated values of its prevalence [5].

According to the present knowledge, lipoedema is a chronic disease and it cannot be fully cured through a conservative approach. Available non-surgical treatment methods focus mostly on preventing the progression of the disease, relieving the patient’s complaints, and increasing the quality of life [14]. Recommended therapy for patients with lipoedema includes compression therapy, manual lymphatic drainage, skin care, intermediate-intensity physical activity, and a healthy diet as conservative treatment and liposuction as surgical treatment [8, 11, 15]. It must be emphasized that liposuction leads to a significant reduction of adipose tissue volume, but in some cases, conservative treatment is still required after surgery [16].

The major problem that women with lipoedema face is a large number of misdiagnosed cases. The low awareness both among medical professionals and the general public leads to an incorrect diagnosis, the condition is usually diagnosed as obesity or lymphoedema [17]. Patients are treated for different ailments for many years, and the correct diagnosis is made in the advanced stage of the disease. Therefore the patient may already have additional complications such as mobility problems, a significant reduction in daily activities, and a high dependence on others [11, 18].

Although it is necessary to distinguish between lipoedema and obesity, excessive body weight in the form of obesity or being overweight is often present in people with lipoedema. According to the current knowledge, there is no sufficient data to conclude that lipoedema causes weight gain or that excess body weight contributes to the development of lipoedema. It is known, that although lipoedema and obesity are not comorbidities, when excessive body weight is present additionally to lipoedema it significantly aggravates the patient’s condition and ability to function independently [11].

The main aim of this study is to present the state of knowledge about lipoedema among Polish women. The purpose is also to explore whether there is an association between BMI, self-reported symptoms, age, and lipoedema awareness among participants.

## Methods

### Participants

A total of 170 participants took part in the study. The respondents were recruited from social media groups for women seeking advice on body weight disorders or wanting to reduce their weight. Data on height and body weight were collected in order to calculate BMI (Body Mass Index). According to World Health Organization, normal body weight is identified when BMI ranges from 18.5 to 24.9 kg/m^2^, being overweight when BMI is between 25 and 29.9 kg/m^2^, and obesity when BMI is above 30 kg/m^2^.

### Methods

The research method was the original anonymous questionnaire conducted online. The study was approved by the Independent Bioethics Committee for Scientific Research at the Medical University of Gdansk (912/2021–2022). The questionnaire created with Google Forms was posted on social media groups and forums for women. A survey study was carried out in January 2022 and was completed by 170 women. Participants filled out the survey form voluntarily and did not require a signature due to the anonymous nature of the online survey. The survey consisted of 12 questions divided into 3 parts. The first part contained 3 questions about the metric data (age, self-reported body weight, and self-reported height), and the second part consisted of 3 questions about the presence of symptoms that may suggest the occurrence of lipoedema. The last part contained 6 questions aiming at assessing the knowledge about the definition of lipoedema, its symptoms, possible treatment methods, and differentiation between obesity and lipoedema. The survey was created by the authors of this study and all of the questions have been formulated according to current knowledge about lipoedema from guidelines and systematic reviews [8, 11, 18–20]. A full version of the created survey is available as a Supplement Material (S1).

### Statistical analysis

Statistical analysis of this study was conducted using Statistica 13.3, and Microsoft Excel. Descriptive statistics were presented with mean and standard deviation, and categorical variables were expressed as a percentage. The Mann-Whitney U test and ANOVA were used to access the significance of the results depending on age, BMI, and number of lipoedema symptoms. *P* value under 0.05 was considered significant.

## Results

### Characteristics of participants

All of the participants were female and their ages ranged from 18 to 68 (the mean was 33.7). The nationality of all of the surveyed women was Polish. The lowest BMI value among all subjects was 18.72 kg/m^2^, and the highest was 45.73 kg/m^2^. The average BMI value was 28.45 kg/m^2^. The BMI values in the majority of participants indicated obesity (*n* = 68, 40%) while being overweight, and normal body weight was reported in the same proportion of the subjects (*n* = 51, 30%).

The respondents were also asked to subjectively report whether they experienced any of the features that may be indicative of lipoedema. Overall, the largest proportion of women included in this study reported having difficulties losing weight through diet and physical activity (44%), 40.6%, 36.5%, and 33.5% reported disproportion between slim upper body and enlarged lower extremities, pain/heaviness in the legs, and increased tendency to bruising, respectively. The occurrence of swelling in lower limbs was indicated by 32% of the respondents, 27.6% reported deposition of adipose tissue mostly around the lower extremities, and 21% had characteristic fat cuffs around the ankles. The least proportion of subjects reported the feet not being affected by oedema (16.5%).

### Lipoedema definition

Overall out of 170 respondents, only 36 women admitted that they knew the term ‘lipoedema’. However, the correct definition of lipoedema was indicated only by 22 women. It can suggest that some subjects were confident about their knowledge of lipoedema, however, in fact, they didn’t recognize the disease. Table 1 presents the correlation between knowledge about lipoedema and the indication of the correct definition. The majority of the respondents admitted that they did not have any knowledge about lipoedema, and their ignorance was confirmed by choosing the incorrect definition. The indication of a correct definition, and the lack of recognition of the term ‘lipoedema’, which occurred in 5.8% of the subjects, was considered random and was excluded from further analysis. 14% of the women admitted that they knew the term lipoedema but they had indicated an incorrect diagnosis. These individuals were considered unaware of their ignorance of the definition of lipoedema. Ultimately, only 7% of the subjects demonstrated knowledge of the definition of lipoedema.

**Table 1:** Knowledge of the term ‘lipoedema’ in correlation with knowledge of its definition

Declaration of the Knowledge of Lipoedema	Correct lipoedema definition	Incorrect lipoedema definition
“I know what lipoedema is”	12 (7%)	24 (14%)
“I do not know what lipoedema is”	10 (6%)	114 (73%)

### Characteristic features

The surveyed women were asked to indicate characteristic features of lipoedema. More than one answer was allowed. Out of all the women-only 20 (12%) were able to indicate all the correct lipoedema symptoms. Even though pain and heaviness in the legs is a characteristic feature of lipoedema, it can also be present in different conditions such for example, venous insufficiency. Indication of this feature by a large number of respondents (61.8%) can be a result of the general association of oedema with this symptom. Therefore, the answer “Affects mostly women” could be indicated because oedema, regardless of the type, is generally thought to be prevalent among the female population [21, 22]. Moreover, women are overall more aware of fluid retention and fluctuations due to the menstrual cycle. An extremely low level of indicating the feature “Symmetric” suggests that the subject’s knowledge of lipoedema is very low since it is one of the most characteristic lipoedema features. All responses to this question are presented in Table 2.

**Table 2:** Knowledge of characteristic features of lipoedema

Feature	Accuracy of the answer	Number of participants indicating certain features (*N* = 170)
Affects mostly women	correct	83 (48.8%)
Symmetric	correct	25 (14.7%)
Pain and heaviness in legs	correct	105 (61.8%)
Asymmetric	incorrect	87 (51.2%)
Feet are the most affected part of the body	incorrect	99 (58.2%)
Lipoedema can occur in every part of the body	incorrect	58 (34.1%)

### Treatment

The survey incorporated two questions on the treatment of lipoedema: one of them was focused on the methods of conservative treatment, and the other one concerned the feasibility of curing lipoedema.

Regarding the first question, the respondents were allowed to mark more than one method of treatment. Only 7 (4%) subjects were able to correctly demonstrate all of the conservative methods of lipoedema treatment. Since lipoedema is still not an entirely understood condition, treatment options remain limited. Methods used to alleviate lipoedema symptoms are usually also employed in treating different types of oedema. Hence, probably a large percentage of the correctly indicated methods of therapy, i.e. Manual Lymphatic Drainage (67.6%), Movement Therapy (60%), and Compression Therapy (55.3%). Table 3 presents all responses to the first question regarding treatment.

**Table 3:** Knowledge of methods of treatment of lipoedema

Method of treatment	Accuracy of the answer	Number of subjects (*N* = 170)
Movement therapy	correct	103 (60%)
Manual Lymphatic Drainage	correct	115 (67.6%)
Compression therapy	correct	94 (55.3%)
Skincare	correct	28 (16.5%)
Electrotherapy	incorrect	36 (21.2%)
High-intensity training	incorrect	24 (14.1%)
Infrared therapy	incorrect	42 (24.7%)

Overall 63 (37%) of respondents correctly stated that the treatment of lipoedema required lifelong conservative therapy and, in some cases, surgical treatment. More than half of the surveyed subjects claimed that lipoedema could be fully cured with a balanced diet and regular physical activity. The fact that the majority of women think that lipoedema can be cured with exercise and diet is quite disturbing because this belief usually leads to downplaying lipoedema.

### Differences between lipoedema and obesity

An incorrect answer was indicated by exactly half of the respondents. Even though 50% of them could identify the basic difference between obesity and lipoedema, it can be assumed that since the answers to all of the other questions suggested lower lipoedema awareness some of the responses could have been random. All of the responses are presented in Table 4.

**Table 4:** Knowledge of the difference between obesity and lipoedema

Question	Accuracy of the answer	Number of subjects
The disproportion between the slim trunk and thickened extremities is not common for obesity unlike in lipoedema	correct	85 (50%)
Tendency to bruising is not present in both lipoedema and obesity	incorrect	45 (25%)
Adipose tissue in lipoedema is accumulated around the abdomen to a greater extent than in obesity	incorrect	40 (25%)

### Overall lipoedema awareness according to age, BMI, and number of experienced symptoms

Our research aimed at exploring the state of knowledge on the topic of lipoedema among women. For this purpose, each participant scored points for correct answers to the above-mentioned questions. The score ranged from 0 to 5 points, and the mean score was 1.1 (*N* = 170, SD = 1.08). The participants were assigned to groups based on the following features age, BMI, and the number of experienced symptoms. The number of correct answers was assessed within the groups. Table 5 presents the summary of the knowledge of lipoedema among surveyed women.

**Table 5:** Summary of the survey results

	Sample		Knowledge summary			
***Characteristics***	***n***	***%***	***Mean***	***SD***	***Significance***	
**Age**						
Below 30	80	47.05%	1.2125	1.1585	P = 0.277308	
Above 30	90	52.95%	1.1	0.9888		
**Symptoms**						
Up to 2	95	55.88%	1.3263	1.06	P = 0.000504	
2 and more	75	44.12%	0.8133	1.03		
**BMI**						
Normal	51	30%	1.156	1.08	P = 0.06	
Overweight	51	30%	1.294	1.02		
Obesity	68	40%	0.9117	1.102		

The results revealed no significant difference in the knowledge depending on age ( below 30 years old and above 31 years old) and BMI (normal body weight, overweight, and obesity). However, a significant difference could be observed depending on the number of symptoms declared by participants. Women who experience 2 or more lipoedema symptoms scored lower (mean = 0.8133) than women with fewer lipoedema symptoms (mean = 1.3263). Along with the increasing number of self-reported symptoms, women may think that these symptoms appear as a result of their frequent body weight changes or they may not associate these symptoms with existing disease.

## Discussion

Lipoedema is a poorly understood condition that only in recent years has been getting more attention. Insufficient knowledge of lipoedema, both among the general public and healthcare professionals, usually leads to misdiagnosis [17]. The exact prevalence remains unknown however various data provide estimated values varying from 7 to 19% of the female population [5, 23–25].

Patients are treated for different conditions and receive lipoedema diagnosis many years after the onset of symptoms [26–28]. In a survey study conducted by Lipoedema UK, out of 250 women with lipoedema, only 9% were diagnosed during the first specialist appointment [27]. Moreover, 44% of the respondents reported being diagnosed by a lymphoedema nurse, 22% NHS hospital consultant, 10% private consultant, 5% general practitioner, 3% MLD specialist, 1% physiotherapist, and 14% reported being diagnosed somewhere else [27].

A study by Romeijn, which involved 163 patients, showed that the average age of onset of the lipoedema symptoms was 20, with lipoedema diagnosed at an average age of 38.3 [28]. In another study involving 209 patients conducted by A.T.Bauer, the first symptoms of lipoedema appeared on average at the age of 16, and the diagnosis was at the age of 31, which shows that, on average, the diagnosis was established after 15 years [26]. According to our study, women indicated various features as characteristics of lipoedema, but only 12% could name them correctly. The selection of the feature “Feet are the most affected part of the body” may suggest that women have mistaken lipoedema for lymphoedema. Accordingly, the indication of the answer “Lipoedema can occur in every part of the body” may suggest the confusion of lipoedema with obesity.

Overall knowledge of lipoedema in our study was low as the correct definition of lipoedema was indicated by 7% of women, and 4%, 12%, and 37% could mark the correct methods of treatment, clinical features, and durability of lipoedema respectively.

Traditionally, conservative treatment of lipoedema is based on Complete Decongestive Therapy, widely used in the treatment of oedemas of different origins [12, 29, 30]. In our study, 67.3% of women chose MLD as lipoedema treatment, and 60%, 55.3%, and 16.5% correctly selected movement therapy, compression therapy, and skin care respectively. Nevertheless, only 4% of respondents were able to indicate all of the conservative methods accurately. In recent studies, however, the usage of some of the elements of CDT is being questioned in lipoedema treatment. Even though MLD is still widely used and recommended for lipoedema patients some authors emphasize that, since in pure lipoedema there is no actual fluid accumulation, recommendation of MLD should be reconsidered [11, 31]. Currently, the application of compression therapy combined with exercises is thought to be the most beneficial element of lipoedema conservative treatment [31, 32]. Moreover, the possibilities of surgical treatment should be highlighted, since liposuction is the only method that enables a significant decrease in the volume of adipose tissue. A study by T. Witte et al. assessed the effects of liposuction among 63 lipoedema patients and the results showed that only 44% of patients still needed conservative treatment after liposuction [25]. Another study by Baumgartner evaluated the effectiveness of liposuction after 4, 8, and 12 years. Out of 37 patients, 27% did not need any component of CDT, 19% reduced the use of conservative treatment, and 54% continued to undergo CDT 12 years after liposuction [33]. Contrary, a study by Conely, showed that out of 592 lipoedema patients, only 3% still needed CDT 15 years after surgery [34].

Results of our study show that more than half of the respondents believe that lipoedema can be fully cured with diet and physical activity. The fact that such a big proportion of participants agree with this statement suggests that they have no understanding of lipoedema, and they consider it to be the same condition as obesity. Recent histologic analysis of lipoedema adipose tissue compared to non-lipoedema tissue showed that there is increased fibrosis and adipocyte hypertrophy in lipoedema tissue, which suggests that pathologic tissue in lipoedema is significantly different from non-lipoedema adipose tissue present in obese patients [8, 35]. Another study assessed the differences in circulating parameters between lipoedema and obese patients. The results showed that there is an increase in LDL cholesterol, oxidative stress, and inflammatory markers and better glucose metabolism among lipoedema patients compared to patients with obesity [36].

Due to a lack of homogenous diagnostic criteria, lipoedema is usually diagnosed by physical examination and patient symptoms [30]. Patients with lipoedema deal with several characteristic ailments. In a study by Dudek showed that out of 98 women with lipoedema, 99% gained weight mostly in legs/arms, 96.8% experienced a feeling of heaviness in the lower extremities, 90.8% reported easy bruising, 86.8% had difficulties losing weight, and 82.6% experienced pain on pressure [37]. Our study of women who were not previously diagnosed with lipoedema showed that 44% of them had difficulties losing weight, 40.6% reported a disproportion between the upper and lower part of the body, 36.5% experienced pain/heaviness in the legs, 33.5% reported easy bruising, and 27.6% stated that accumulation of fat was mostly in lower extremities. Those results demonstrate that some portion of surveyed women reported having features characteristic of lipoedema.

The accurate diagnosis of lipoedema should definitely be preceded by clinical examination and anamnesis, but the aim of our study was an assessment of knowledge of lipoedema among women, not a diagnosis. Moreover, the willingness to fill out the questionnaire voluntarily by these women may also prove that this group of women is looking for an explanation of their health problem by visiting various websites, forums, and support groups.

### Implications for practice and/or policy

Our research may help spread knowledge about lipoedema both among medical professionals and the general population, which could have an impact on decreasing the number of undiagnosed cases.

We believe that our work will encourage more researchers to conduct their research on the topic of lipoedema. Increasing the interest in this field is crucial to fully understanding the etiology, pathophysiology, and genetics of lipoedema. As a consequence, it could lead to the development of homogenous diagnostic criteria and appropriate treatment methods.

### Study limitations

The study in the form of an online survey itself has some limitations. We tried to eliminate the risk of the randomness of the answers by special verifying questions, however, due to the character of the online survey study some of the answers may have been guessed by participants. Moreover, the experienced lipoedema symptoms and metric data were self-reported by participants and were not supported by clinical examination. The survey was based on currently available guidelines, however, validation should be considered in the forthcoming studies. The study group was relatively small and specific (Polish women recruited from social media groups), so future research should include a larger proportion of participants.

## Conclusions

The state of knowledge about lipoedema among women is relatively low. Only a few percent of participants were able to correctly answer questions regarding lipoedema. Almost half of the participants reported experiencing 3 or more lipoedema symptoms. The results indicate that this disease is poorly understood and women are looking for an opportunity to participate in research on lipoedema. Improving the situation of people suffering from lipoedema would require targeted information programs that would contribute to earlier diagnosis of the disease, disseminating knowledge about this condition, and thus improving the health of patients. Since this study was relatively small and had a very specific group of participants future research should cover a broader spectrum of participants. Therefore our focus was mostly on a conservative approach, future research should also more extensively address the awareness of surgical treatment options.

### Supplementary Information

**Additional file 1.**

## Data Availability

The datasets used and/or analyzed during the current study are available from the corresponding author upon reasonable request.

## Abbreviations

BMI: Body Mass Index
CDT: Complete Decongestive Therapy
MLD: Manual Lymphatic Drainage
