# Supplementary material for: Assessment of lipoedema awareness among polish women- online survey study
Source: BMC Womens Health. 2023 Aug 29;23:457. doi: 10.1186/s12905-023-02614-7 (PMC10464337; doi:10.1186/s12905-023-02614-7)
Supplement: Supplementary file 1 — Additional file 1. [file 12905_2023_2614_MOESM1_ESM.docx]

Supplementary Material 1

**Lipoedema awareness among women-survey**

1. Age
2. Bodyweight
3. Height
4. Have you ever attempted to lose weight?

A)Yes

B)No

1. Have your attempts to lose weight by dieting and physical activity have been successful?
2. Yes
3. No
4. Please mark all of the symptoms that you experience
5. The disproportion between slim upper body and thicker lower extremities
6. Swelling in lower limbs
7. Pain/heaviness in legs
8. Increased tendency to bruising
9. The fat cuff around the ankles
10. Deposition of adipose tissue mostly around lower extremities
11. Feet are not affected by oedema
12. None
13. Do you know the term ‘lipoedema’?
14. Yes
15. No
16. Please mark the correct definition of lipoedema

A) Chronic disease resulting from excessive fatty tissue growth and significant weight gain, greatly increases the risk of diseases such as m.in diabetes, hypertension, cancer

B) A condition characterized by weight gain and an increase in fatty tissue volume, untreated leads to lipohypertrophy.

C)Progressive disease with excessive symmetrical fat tissue accumulation occurring as a result of hormonal disorders

D)Swelling of tissues caused by lymph stasis

9. Please select 3 characteristic features of lipoedema

A)It mainly affects women

B)Asymmetrical

C)Symmetrical

D)The feet are usually the most affected part of the body

E)Pain and heaviness in legs

F)Lipoedema can occur in every part of the body

1. Please indicate the physiotherapy methods used to treat lipoedema (several answers may be selected)

A)Movement therapy

B)Manual Lymphatic Drainage

C)Compression therapy

D)Skincare

E)Electrotherapy

F)Infrared therapy

G) High-intensity training

1. Please indicate the correct statement concerning the treatment of lipoedema

A)Treatment requires long-term, often lifelong physiotherapy and in some cases surgical treatment

B)Lipoedema can be fully cured by physical activity and diet

C)Treatment consists of the use of diuretics

1. Please indicate the correct sentence regarding lipoedema and obesity

A)Disproportion between slim trunk and thickened extremities is not common for obesity unlike in lipoedema

B)Tendency to bruising is not present in both lipoedema and obesity

C)Adipose tissue in lipoedema is accumulated around abdomen to greater extent than in obesity
